# An Automatic Image Processing System for Glaucoma Screening

**DOI:** 10.1155/2017/4826385

**Published:** 2017-08-29

**Authors:** Ahmed Almazroa, Sami Alodhayb, Kaamran Raahemifar, Vasudevan Lakshminarayanan

**Affiliations:** ^1^Kellogg Eye Center, University of Michigan, 1000 Wall St, Ann Arbor, MI 48105, USA; ^2^King Abdullah International Medical Research Center, King Saud bin Abdulaziz University for Health Sciences, National Guard, Riyadh 14611, Saudi Arabia; ^3^Bin Rushed Ophthalmic Center, King Fahd Branch Rd, Opposite King Fahad National Library, Al Olaya, Riyadh 12311, Saudi Arabia; ^4^Department of Electrical and Computer Engineering, Ryerson University, 350 Victoria St., Toronto, ON, Canada M5B 2K3; ^5^School of Optometry and Vision Science, University of Waterloo, 200 Columbia St. W., Waterloo, ON, Canada N2L 3G1

## Abstract

Horizontal and vertical cup to disc ratios are the most crucial parameters used clinically to detect glaucoma or monitor its progress and are manually evaluated from retinal fundus images of the optic nerve head. Due to the rarity of the glaucoma experts as well as the increasing in glaucoma's population, an automatically calculated horizontal and vertical cup to disc ratios (HCDR and VCDR, resp.) can be useful for glaucoma screening. We report on two algorithms to calculate the HCDR and VCDR. In the algorithms, level set and inpainting techniques were developed for segmenting the disc, while thresholding using Type-II fuzzy approach was developed for segmenting the cup. The results from the algorithms were verified using the manual markings of images from a dataset of glaucomatous images (retinal fundus images for glaucoma analysis (RIGA dataset)) by six ophthalmologists. The algorithm's accuracy for HCDR and VCDR combined was 74.2%. Only the accuracy of manual markings by one ophthalmologist was higher than the algorithm's accuracy. The algorithm's best agreement was with markings by ophthalmologist number 1 in 230 images (41.8%) of the total tested images.

## 1. Introduction

As the world's population has drastically increased, the number of people suffering from glaucoma, or those suspected to have glaucoma, has increased too. Therefore, there is an even greater need for proper diagnosis and effective control of glaucoma. Accurate diagnosis of glaucoma requires three different sets of examinations: (1) evaluation of the intraocular pressure (IOP), (2) evaluation of the visual field, and (3) evaluation of the optic nerve head [1]. Since both elevated-tension glaucoma and normal-tension glaucoma may or may not increase the IOP, the IOP by itself is not a sufficient screening or diagnosis method [2]. On the other hand, visual field examination requires special equipment which is usually available only in tertiary care hospitals equipped with a fundus camera, parametric instrumentation, and possibly an optical coherence tomography [2]. The optic nerve head examination (cup to disc ratio) is a valuable method for diagnosing glaucoma structurally [3]. Primary open angle glaucoma is causing a progressive optic neuropathy and its development is associated with loss of tissue in the neuroretinal rim of the optic disc and that will lead to increase in the size of the optic cup. The pattern of neuroretinal rim loss and cup enlargement may take the form of focal or diffuse change, or both in combination. Focal change, with the loss of the physiological shape of the neuroretinal rim, is identified by careful clinical examination. Diffuse change, with maintenance of the physiological rim shape, is much more difficult to identify. It is in these cases that quantification of the neuroretinal rim area or cup size is useful. Methods have been described to estimate the area of the neuroretinal rim during ophthalmoscopic examination, but several measurements and calculations or additional equipment are required. Clinical estimation of the size of the cup using either the slit lamb or a simple imaging modalities such as fundus images is a significant clinical parameter and remains the simplest and most frequently performed assessment of the optic disc in the diagnosis and follows up the progression of the glaucoma suspect. The estimation of the size of the cup is usually made by comparison with the size of the disc and given as the ratio of the vertical and horizontal diameter of the cup to the vertical and horizontal diameter of the disc based on Garway-Heath et al. [4]. Thus, an automatic system for examination of optic nerve head is very useful. In a recent paper, Almazroa et al. [5] critically review the literature on glaucoma image processing.

Recently Dhumane and Patil [6] have developed an algorithm for calculating the cup to disc ratio. In this algorithm superpixel segmentation was used to extract disc and boundaries. Thirty-seven images were used to test the algorithm and it successfully segmented 33 images. Guerre et al. [7] introduced a technique based on Otsu's adaptive thresholding and a support vector machines classifier with linear kernel. The algorithm was tested on two datasets (29 and 26 images), and the accuracies of the cup to disc ratio were 89% and 59%, respectively. Zilly et al. [8] proposed a novel convolutional neural network based method for optic cup and disc segmentation. To reduce computational complexity, an entropy based sampling technique was introduced. The algorithm was tested using 10 images and the overlap was 89.5% between the segmented disc and ground truth, and 86.4% between the segmented cup and ground truth. Issac et al. [9] introduced a technique based on adaptive thresholding using features from the image such as mean and standard deviation. The algorithm was tested on 63 images and the accuracy was 92.06%. Alghmdi et al. [10] developed an automatic system to measure the cup to disc ratio based on superpixels clustering algorithm using simple linear iterative clustering and a feed-forward neural network classifier. The algorithm was tested using 60 images and the mean nonoverlapping error was 11% for the disc and 29% for the cup.

This paper gives the results from calculations of the horizontal and vertical cup to disc ratios using our previously introduced optic disc [11] and cup [12] algorithms. The algorithms were tested using the RIGA dataset. The rest of the paper is organized as follows. The methodology of the research is explained in Section 2. Results are presented in Section 3. We discuss the results and conclude in Section 4.

## 2. Methodology

### 2.1. Dataset

RIGA dataset was collected in order to facilitate research on computer-aided diagnoses of glaucoma. The dataset consists of 750 color fundus images obtained from three different resources: (1) 460 images from MESSIDOR images dataset [13] with two images of sizes 2240 × 1488 pixels and 1440 × 960 pixels, (2) 195 images from Bin Rushed Ophthalmic center in Riyadh, Saudi Arabia. They were acquired in 2014 using a Canon CR2 Nonmydriatic digital retinal camera (less resolution images). The images sizes are 2376 × 1584 pixels. An additional 95 images were obtained from Magrabi Eye Center in Riyadh, Saudi Arabia. The images were acquired between 2012 and 2014 using a TOPCON TRC 50DX mydriatic retinal camera (more resolution images). The images sizes are 2743 × 1936 pixels. The images were notated manually by 6 ophthalmologists individually. Each one notated the disc and cup boundaries manually using a precise pen for Microsoft surface pro 3 with 12 inches high resolution screen (2160 × 1440 pixels). Six parameters were calculated for the manual marking in order to be used to evaluate the algorithms, namely, disc area, disc centroid, cup area, cup centroid, vertical cup to disc ratio, and horizontal cup to disc ratio. The 3 datasets contain both normal and glaucomatous fundus images.

The dataset was divided into two sets: training set with 200 images and testing set with 550 images for the training and testing purpose for the developed algorithms (Table 1).

### 2.2. Optic Disc and Cup Segmentation

**Figure 1:**
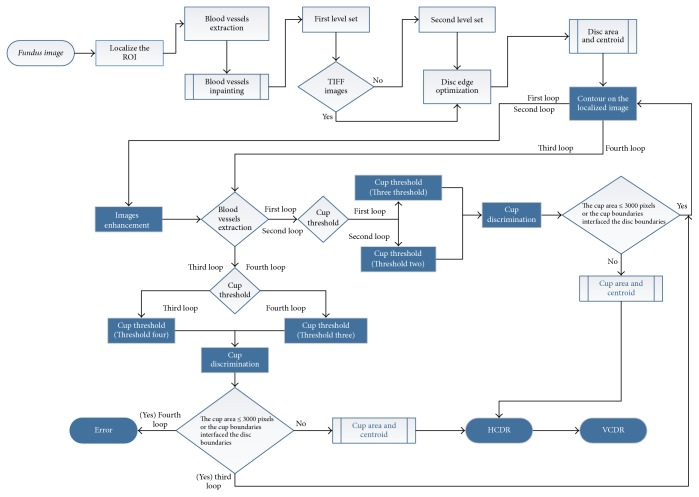
The final algorithm flowchart.

Briefly, the optic nerve head was localized using the procedures explained by Almazroa et al. [14] and Burman et al. [15] and optic disc segmentation was introduced by Almazroa et al. [11] based on inpainting the blood vessels and level set method. A fast digital image inpainting technique [16] was applied. The blood vessels were extracted; thus the extracted blood vessels are utilized to be the mask which identifies the area that wants to be inpainted. Blood vessels were extracted using a top-hat transform on the G-channel of the fundus image. In the second step, the segmentation process represented by the active contour model implemented by the level set [17] was applied. Based on the quality of the image, one of the two paths was considered for applying the level set (Figure 1). From the three sets of images in RIGA dataset, Bin Rushed images are low quality and need a double level set. After applying the first level set, the contour was considered as a second optic disc localization in order to restrict the variations from the center that cause the problems. Then the second localization was split into two to apply the level set again in order to obtain a more accurate segmentation.

On the other hand, the cup segmentation was introduced by Almazroa et al. [12]. The blood vessels were extracted using the same approach as that used for optic disc segmentation. Image thresholding was applied using an Interval Type-II fuzzy entropy based thresholding scheme with Differential Evolution on the localized image to detect the intensity of the optic cup borders. Hough transform was used to approximate the cup boundaries.

Four loops were considered for four different threshold values based on some conditions which will be discussed later.

The training images were the keys for developing this algorithm. The aforementioned thresholding technique was used in order to detect the cup boundaries based on the image intensity. Since the cup shape and structure are not constant among the people either for normal or for glaucomatous cases, that leads to making the boundary segmentation process more complicated. Using the thresholding technique, the image will be thresholded into binary image (black and white); that is, if threshold value (1) is applied (simple threshold concept), and three image intensities if threshold value (2) is applied and so forth. Therefore, different threshold values starting from 1 up to 30 were applied using the 200 training images in order to find out the comprehensive threshold value which will detect the cup boundaries for different images sizes and quality as well as different cup cases. The 200 training images with the six ophthalmologists manual annotations were the guide for choosing the best threshold value. Therefore, four loops for the cup segmentation algorithm with four different threshold values as shown in Figure 1 were decided to be the best values to segment the cup boundaries.

In more detail, as a first step threshold value (3) was chosen in the first loop, that is, dividing the image intensity into four parts. Then, the cup was chosen as the brightest spot. However, occasionally some small bright spots appeared too, which do not belong to the cup; therefore, white spots of less than 50 pixels were eliminated to reduce the chance of errors when selecting the cup. Then the image was converted to binary after deciding about the brightest spot (representing the cup). There were still some small gaps in the selected white spot due to the removed blood vessels. Therefore, the extracted blood vessels were brought back in order to fill out the gaps between the white spots.

A morphological closing operation was applied to close the small gaps that remained in the white spots even after adding the blood vessels in order to prevent errors that might occur when applying the last step of segmenting the cup (Hough transform). As a result, the cup area and centroid are then calculated.

The decision for applying the second, third, and fourth loop will be automated and based on some conditions. If the calculated cup area is equal to or less than 3000 pixels or the contour of the segmented cup boundaries touches the already segmented disc boundaries, then that will lead to error and the second loop will be run. Otherwise, there is no error and then the VCDR and HCDR will be calculated based on the disc and cup segmented boundaries.

In case of error, threshold (2) is then applied (three intensities) as the second loop as shown in the flowchart in Figure 1 with considering the aforementioned conditions of the first loop in order to decide whether there is an error or no. If there is an error, then the third loop will be applied; otherwise the VCDR and HCDR will be calculated.

The same conditions were considered for the third loop. However, in the third loop threshold value (4) was applied (five intensities). Though, unlike the first two loops, in this loop there was no image enhancement (Figure 1). If the segmentation for the third loop matched the aforementioned conditions, then the final loop is applied for threshold value (3) (four intensities) and also without image enhancement.

In conclusion, for any loop, the cup area and centroid are calculated when the process did not pass the two conditions. The postprocessing in the all algorithm was calculating the horizontal cup to disc ratio (HCDR) and the vertical cup to disc ratio (VCDR).

## 3. Results

### 3.1. Horizontal Cup to Disc Ratio (HCDR)

To calculate the HCDR using the manual marking of the disc and cup, the furthest two pixels horizontally were considered for the disc and cup separately, and then their ratio was calculated.

The same procedure was followed for automatic calculation of HCDR after segmenting the disc and cup. Three parameters' outliers were considered in order to filter the images for the HCDR [14]: (1) the disc outliers (area and centroid), (2) the cup outliers (area and centroid), and (3) the HCDR outliers. Those parameters were chosen in order to calculate accuracy between the six ophthalmologists; then the filtered images are used to evaluate any developed algorithm. Firstly, the standard deviation (SD) between the 6 ophthalmologists for every fundus image was calculated for the disc (area and centroid) and cup (area and centroid) separately. Secondly, a mean SD was calculated for every parameter which will be the judge between the six ophthalmologists for every parameter, that is, deciding whether there is an outlier (when SD of the image > mean SD) between the ophthalmologists in marking of the disc or cup boundaries by testing the six manual markings one by one. The mean and standard deviation for all the disc and cup parameters were different based on the different size of the images, which will affect the size of the disc and cup for every images dataset; more details are explained in [14].

**Figure 2:**
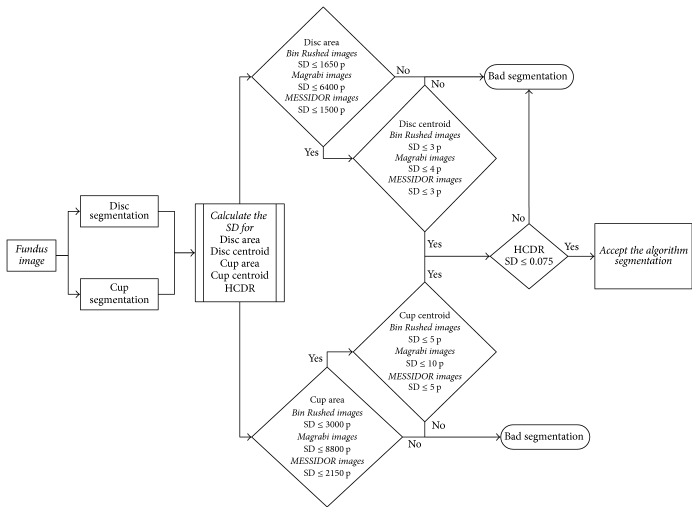
The flowchart for HCDR calculation.

Thirdly, the mean SD for the HCDR was 0.075. Any manual marking making the HCDR SD more than 0.075 was considered as an outlier and was eliminated from further analysis. The same was true for the automated system (Figure 2). Thus, many images were eliminated. Removing the outliers obviously affects the number of agreements in markings done by the ophthalmologists as well as the algorithm. Therefore, three parameters were considered when deciding whether an image could be used for evaluation of the algorithm. If there were at least three outliers for a certain parameter, for example, disc area, for an image, then the image was eliminated from evaluating the algorithm. While based on the statistical analysis in [14] if there were three outliers from different parameters for one image, for example, one for disc centroid, one for cup area, and one for HCDR, then the image was not eliminated. However, if there were four outliers with two of them on the same parameter, for example, two outliers for the disc area, one for the cup area and one for the HCDR, then the image was eliminated. That will be including all the images either normal or glaucomatous. Filtering the images based on the aforementioned method, the verity of the manual marking of the disc and cup boundaries among the 6 ophthalmologists will be considered as unclear diagnosis whether for normal or glaucomatous images which will be leading to removing the outlier images from evaluating the new algorithm.

**Figure 3:**
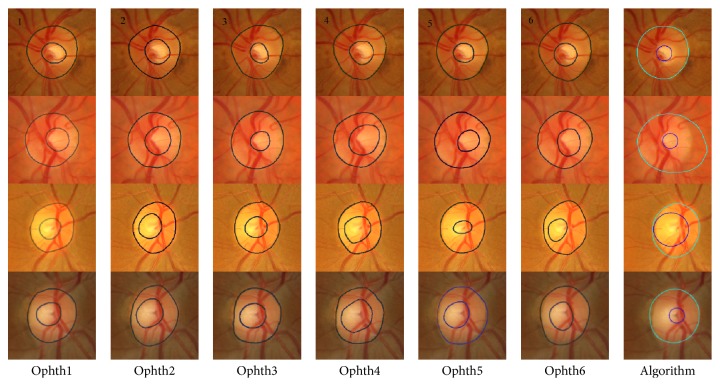
Examples of poor segmentation results for horizontal cup to disc ratio. Each row shows the results for a sample image. The images on each row (left to right) show the results of manual markings by the six ophthalmologists (1 to 6) and the automatic marking by the algorithm (far right).

Figure 3 shows the bad segmentation results for the HCDR for four different images each with a different condition. For the first image, represented in the first row, marking by ophthalmologist number six was eliminated due to the disc area which affected the HCDR, while the algorithm gave bad results due to the cup size. In the second image, represented in the second row, the markings by ophthalmologists numbers four and six were eliminated because of the cup area and centroid, respectively. The algorithm gave bad results due to bad disc segmentation. In the third image, the markings by three ophthalmologists were eliminated for different reasons; therefore, this image was not considered in evaluation of the algorithm. In the fourth image, the markings by ophthalmologists numbers four and six were eliminated due to the cup area and disc centroid, respectively. The algorithm gave a bad result due to the bad cup area segmentation.

**Figure 4:**
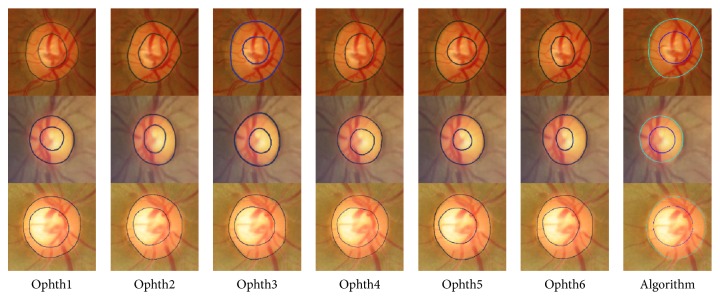
Examples of good segmentation results for horizontal cup to disc ratio. Each row shows the results for a sample image. The images on each row show the results of manual markings by the six ophthalmologists (1 to 6; left to right) and the automatic marking by the algorithm (far right).

Figure 4 shows examples of good segmentation results for HCDR. In the first image, represented in the first row, the algorithm gave good results for the disc, cup, and HCDR for MESSIDOR dataset where the SD was 0.04 between the six ophthalmologists and became 0.055 when the algorithm results were included (still less than the mean SD (0.075)). Furthermore, the HCDR given by the algorithm was 0.58, while it was reported to be 0.54, 0.55, 0.51, 0.56, 0.55, and 0.45 by ophthalmologists numbers one to six, respectively. In the second image, shown in the second row, the algorithm gave good result for Bin Rushed dataset where the SD was 0.06 between the six ophthalmologists and became 0.065 when the algorithm results were included. Furthermore, the HCDR given by the algorithm was 0.58, while it was reported to be 0.54, 0.51, 0.49, 0.54, 0.43, and 0.40 by ophthalmologists numbers one to six, respectively. Finally, in the last image, the algorithm gave good results for Magrabi dataset where the mean SD was 0.03 and became 0.025 when the algorithm results were included. Furthermore, for this dataset the HCDR given by the algorithm was 0.68, while it was reported to be 0.69, 0.66, 0.70, 0.73, 0.68, and 0.66 by ophthalmologists numbers one to six, respectively.

#### 3.1.1. Results of Bin Rushed Dataset

As can be seen in Table 2, the algorithm achieved 73.8% accuracy, the second best accuracy, when testing a total of 111 images. In total, 84 images were eliminated; 74 of them had at least three outliers (manual marking) in disc, cup, or HCDR calculations and 10 images were not localized. Ophthalmologist number one had the best performance in calculating HCDR, achieving 79.6% accuracy for the 108 total tested images. The accuracies of performances of the six ophthalmologists as well as the algorithm were in the short range of 74–86 images. However, the total number of tested images had obviously affected the accuracy.

#### 3.1.2. Results of Magrabi Dataset

Fewer images from Magrabi dataset were used to evaluate the algorithm. As shown in Table 3, in total 95 images were used from which 6 images were not localized and 31 to 43 images were eliminated due to the outliers in manual markings of disc, cup, or HCDR. For this image set, ophthalmologist number two had the best performance, testing 51 images in total with the accuracy of 39 images. The performance of the algorithm was the second best. The total number of images tested by the algorithm was 46 images. Most of the outliers that resulted in eliminating the images were due to errors in markings of ophthalmologists numbers six, three, and five.

#### 3.1.3. Results of MESSIDOR Dataset

Finally, the algorithm was tested on MESSIDOR dataset containing 260 images (Table 4). Here 10 images were not localized. Furthermore, 63 to 73 images were eliminated from further analysis since their manual markings were outliers. Ophthalmologist number one had fewer outlier markings, that is, only in 56 images. The best accuracy result was obtained by ophthalmologist number three based on testing 177 images; the accuracy was 143 images. The accuracy of markings by ophthalmologist number one was 76.2%; that is, 148 images were accurately marked. The algorithm was the third best in terms of accuracy; from 186 images tested, 139 were accurately marked.

#### 3.1.4. Consolidated Results for HCDR

The final results for all three datasets are reported in Table 5. With 269 images accurately marked, markings by ophthalmologist number one had the highest percentage of accuracy (76.6%). The algorithm was the second best with 74.6% accuracy and 256 images accurately segmented. Markings by ophthalmologist number 4 had the most outliers. The number of eliminated images ranged from 161 images for ophthalmologist number four to 181 images for the algorithm.

**Figure 5:**
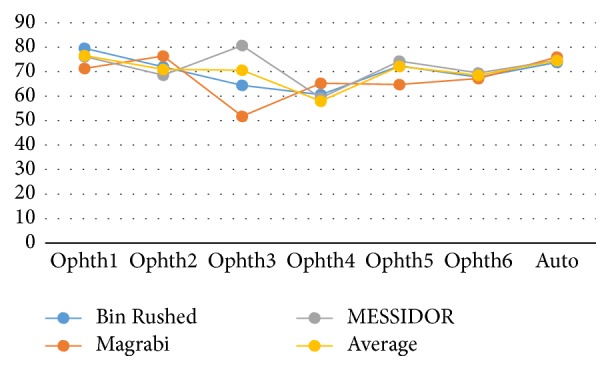
The percentage accuracy of the HCDR for the three image sets separately and together.

Figure 5 illustrates the variation in percentage of accuracy among the three datasets. Ophthalmologist number six and the algorithm showed the same results for all images despite their differences in quality and size. However, the performance of ophthalmologist number three varied significantly; he showed greatest accuracy while working on MESSIDOR dataset and the lowest accuracy while working on Magrabi dataset.

#### 3.1.5. Agreement for HCDR

The best agreement was observed between the markings by ophthalmologists numbers one and five, where the markings were in 251 of 550 images (45.6%) (Table 6). The best agreement for the algorithm was with markings by ophthalmologist number one (agreement in 239 images (43.4%)). On the other hand, the lowest agreement was observed between markings by ophthalmologist number four and the algorithm (agreement in 162 images (29.4%)).

In terms of the total number of image agreements, the algorithm was in the sixth place, which does not correspond with its ranking in accuracy.

**Figure 6:**
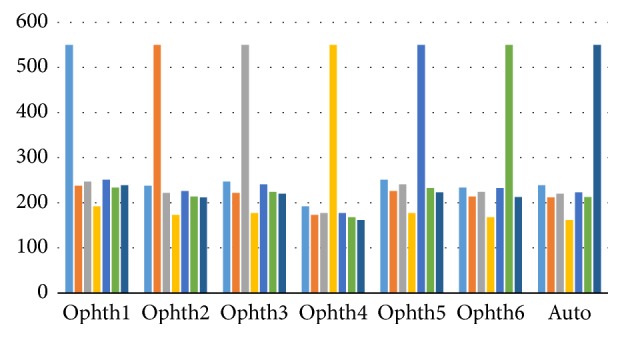
The number of images agreed for the HCDR between the ophthalmologists and the algorithm. *x*-axis represents the number of 6 ophthalmologists and the algorithm. *y*-axis represents the number of agreed images.

Figure 6 clearly illustrates that ophthalmologists numbers two, three, four, and five and the algorithm were in best agreement with ophthalmologist number one. Ophthalmologists numbers three and six had the best agreement with ophthalmologists numbers one and five.

The number of images agreed upon for all six ophthalmologists as well as the algorithm ranged from 1250 to 1400 in total, except for ophthalmologist number four (Table 5).

### 3.2. Vertical Cup to Disc Ratio (VCDR)

**Figure 7:**
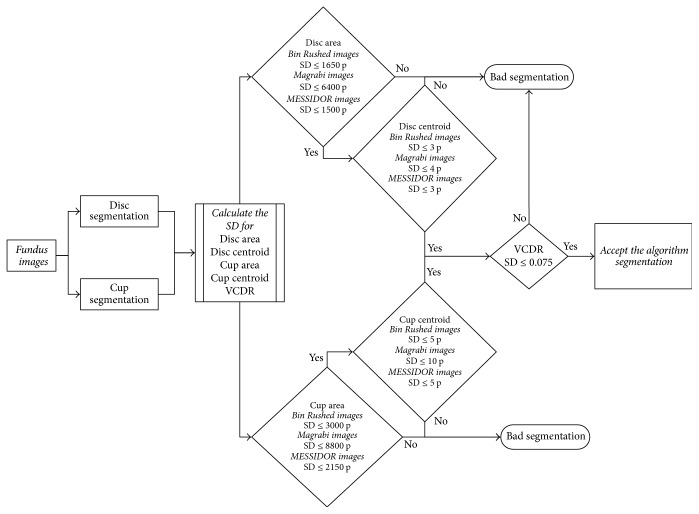
Flowchart of the analysis of VCDR calculation.

The procedures used for calculation of HCDR were repeated to calculate VCDR for the algorithm. Two parameters were considered: disc (area and centroid) and cup (area and centroid). The procedures for eliminating the outliers were the same as those used in HCDR analysis and followed the same steps as shown by Almazroa et al. [14]. The same procedures were also conducted for the algorithm in order to decide whether the segmentations were accepted or not (Figure 7).

Three parameters were considered in order to decide whether an image could be used to evaluate the algorithm. For each image, if there were at least three outliers from the same parameter, the image was not used for evaluating the algorithm. However, if the three outliers were from different parameters, for example, one for disc centroid, one for cup area, and one for VCDR, then the image was not eliminated. On the other hand, if there were four outliers from two different parameters, for example, two outliers for the disc area, one outlier for the cup area, and another for the VCDR, then the image was eliminated from the evaluation.

**Figure 8:**
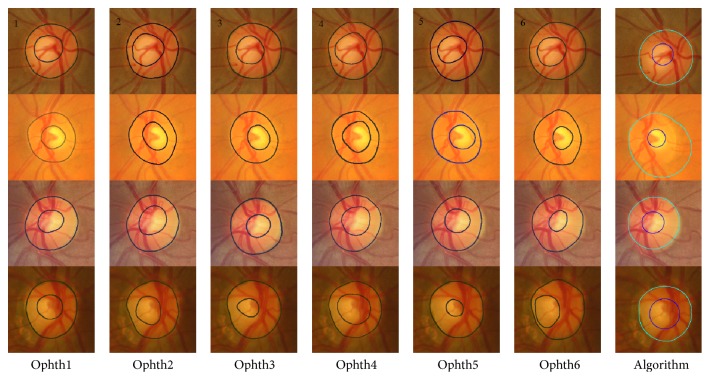
Examples of poor segmentation results for vertical cup to disc ratio. Each row shows the results for a sample image. The images on each row show the results of manual markings by the six ophthalmologists (1 to 6; left to right) and the automatic marking by the algorithm (far right).

Figure 8 shows examples of poor segmentation results for HCDR. Each row shows the results for a sample image. The images on each row show the results of manual markings by the six ophthalmologists (one to six) and the automatic marking by the algorithm. In the first image, represented in the first row, marking by ophthalmologist number one was removed because of the disc area, and marking by ophthalmologist number six was removed because of the disc area and cup centroid. The algorithm gave bad results of the VCDR due to bad cup area. In the second image, markings by ophthalmologists numbers two and four were eliminated because of bad cup area, and the algorithm gave bad results due to bad disc segmentation. In the third image, markings by ophthalmologists numbers three and six were removed due to the cup area and centroid. The algorithm gave good results in terms of disc and area. However, the mean SD of VCDR was beyond the 0.075 threshold and therefore was considered an outlier. Hence, this result was considered inappropriate for calculating the accuracy. In the last image, there is clearly a big variation among manual markings; therefore, this image was not considered a good image for evaluating the algorithm.

**Figure 9:**
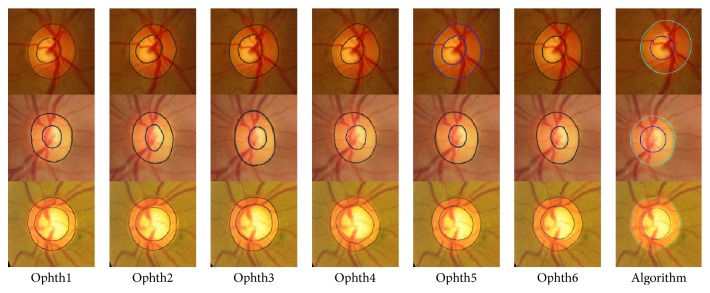
Examples of good segmentation results for vertical cup to disc ratio. Each row shows the results for a sample image. The images on each row show the results of manual markings by the six ophthalmologists (1 to 6) and the automatic marking by the algorithm.

Figure 9 shows examples of good segmentation results for VCDR. Each row shows the results for a sample image. The images on each row show the results of manual markings by the six ophthalmologists (1 to 6) and the automatic marking by the algorithm. For the first image (presented in the first row) the SD was 0.03 for the six ophthalmologists and 0.04 when the algorithm's results were included. The VCDR was 0.45 for the algorithm while it was 0.46, 0.52, 0.54, 0.50, 0.54, and 0.48 for ophthalmologists numbers one to six, respectively. For the second image, the SD was the same for all manual markings and the algorithm, and its value was 0.04.

Here the VCDR was 0.52 for the algorithm and 0.47, 0.52, 0.41, 0.49, 0.42, and 0.44 for ophthalmologists numbers one to six, respectively. In the last image, the SD was 0.03 for the six ophthalmologists and 0.04 with the algorithm result. The VCDR was 0.61 for the algorithm and 0.63, 0.63, 0.67, 0.70, 0.64, and 0.62 for ophthalmologists numbers one to six, respectively.

#### 3.2.1. Results of Bin Rushed Dataset

The accuracy of the VCDR for all markings by the six ophthalmologists as well as the algorithm for Bin Rushed dataset is shown in Table 7. Sixty-four to eighty images were eliminated from markings by the ophthalmologists as well as the algorithm due to the many outliers for either disc, cup, or the VCDR. Ophthalmologist number one was the best in terms of the percentage accuracy; the algorithm was the second best followed by ophthalmologist number six. In terms of the number of images accurately marked, ophthalmologist number one marked 91 out of 107 images accurately, followed by the algorithm which segmented 82 out of 109 images accurately and then ophthalmologist number six who accurately marked 79 out of 105 images.

#### 3.2.2. Results of Magrabi Dataset

The percentage accuracies for Magrabi dataset are shown in Table 8. Thirty-eight to forty-four images were eliminated from the markings by the ophthalmologists as well as the algorithm. The best percentage accuracy belonged to the markings by ophthalmologist number six. The algorithm was the third best in terms of percentage accuracy. In terms of the number of images accurately marked, ophthalmologists numbers two and six and the algorithm had the highest performance with 38 out of 49, 48, and 50 images (respectively) marked accurately.

#### 3.2.3. Results of MESSIDOR Dataset

Finally, the percentage accuracy for MESSIDOR dataset is shown in Table 9. Sixty to 81 images were eliminated from the work of all six ophthalmologists as well as the algorithm. These numbers were similar to the number of images eliminated from this dataset for calculation of HCDR which were between 56 and 73 images. Ophthalmologist number three had the best performance in terms of percentage accuracy, while the algorithm was at fifth place. In terms of the number of images, ophthalmologist number three had 149 out of 169 images marked accurately, while the algorithm successfully segmented 138 out of 186 images.

#### 3.2.4. Consolidated Results for VCDR

Table 10 shows the final results for the VCDR. One hundred seventy to 197 images were eliminated due to outliers from the work done by all six ophthalmologists as well as the algorithm (for HCDR, 161 to 181 images were eliminated due to outliers). The best percentage accuracy was 79.2% and belonged to the 332 images tested by ophthalmologist number one. For HCDR the best percentage accuracy was 76.6% and belonged to the 351 images also tested by ophthalmologist number one. The algorithm percentage accuracy was the fourth best with 74.7% of 345 total tested images (for the HCDR the algorithm percentage accuracy was the second best with 74.4% of 343 total tested images). In conclusion, for VCDR the markings by the ophthalmologists and the algorithm had similar results in terms of the total tested images as well as percentage accuracy, while for HCDR the algorithm gave better results than manual markings.

**Figure 10:**
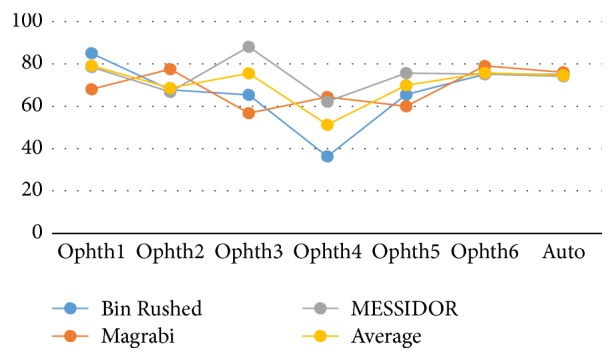
The percentage accuracy of the VCDR for the three images set individually.

Figure 10 shows how the six ophthalmologists as well as the algorithm performed (in terms of VCDR percentage accuracy) on the three datasets. The algorithm had consistent performance regardless of the dataset. This finding was similar to what we observed for the HCDR. The performance of ophthalmologist number six also remained the same across the three datasets. Similar to what was observed for the HCDR, there were small variations in performance of ophthalmologists numbers one, two, and five as they worked on different datasets, and ophthalmologist number three showed the most variable performance. Finally, while ophthalmologist number four showed a rather consistent performance for HCDR, his performance for VCDR showed big variation.

#### 3.2.5. Agreement for VCDR

Table 11 shows the agreements between the ophthalmologists and the algorithm in terms of the number of images. The best agreements were between ophthalmologists numbers one and six in 254 images (46.1%), then between ophthalmologists numbers one and three in 253 images (46%), and between ophthalmologist number one and the algorithm in 252 images (45.8%) of 550 images. The agreement among the ophthalmologists regarding VCDR was almost equal to their agreement regarding HCDR. On the other hand, for the VCDR the algorithm was best agreed in 252 images, while for HCDR the algorithm was best agreed in 239 images (43.4%).

**Figure 11:**
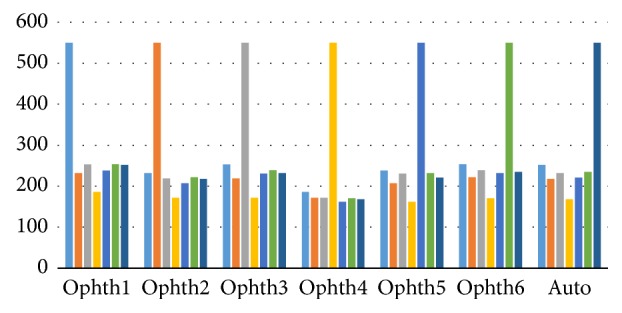
The number of images agreed for the VCDR between the ophthalmologists and the algorithm. *x*-axis represents the number of 6 ophthalmologists and the algorithm. *y*-axis represents the number of agreed images.

Figure 11 shows how the ophthalmologists agreed in the number of images as groups. All six ophthalmologists as well as the algorithm agreed with each other on more than 200 images, except for ophthalmologist number four who agreed with others in less than 200 images.

Ophthalmologist number one had the best agreement with the others.

The range of the total number of images agreed upon was from 1250 to 1400 images, except for ophthalmologist number four who had only 1000 images (Table 10). The range of the total images agreed for the VCDR was similar to the results for HCDR.

### 3.3. Final Results (HCDR and VCDR)

**Figure 12:**
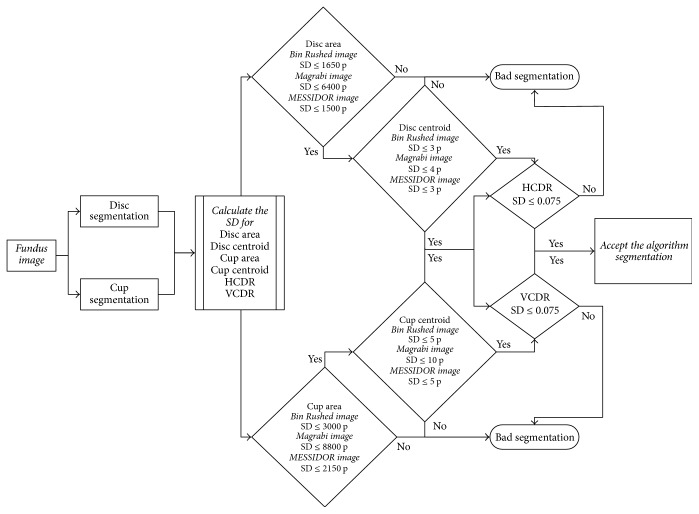
Flowchart for the algorithm analysis.

In this section the analysis covers all four parameters; these are disc (area and centroid), cup (area and centroid), HCDR, and VCDR. This means the manual markings had to pass with respect to all four parameters in order to be included in the analysis [14]. For each image, if there were at least three outliers for the same parameter, for example, three outliers in disc area, then the image was eliminated from evaluating the algorithm. However, if the three outliers were from different parameters, for example, one for disc centroid, one for cup area, and one for HCDR, then the image was not eliminated. If there were four outliers with two outliers belonging to the same parameter, for example, two outliers for the disc area, one outlier for the cup area, and one for the VCDR, then the image was eliminated. The same procedures were applied to the results of the algorithm in order to decide whether a segmentation was accepted or not (Figure 12).

**Figure 13:**
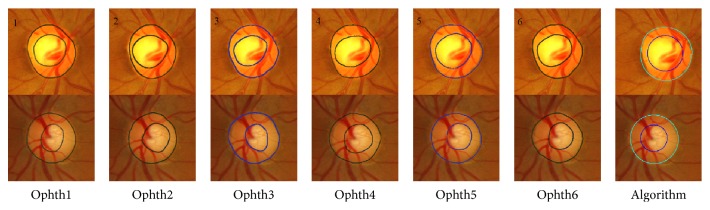
The algorithm final results for MESSIDOR images set.

Figure 13 shows the results for two images from the MESSIDOR dataset. The HCDRs recorded by ophthalmologists numbers one to six were 0.67, 0.76, 0.67, 0.73, 0.69, and 0.70, respectively, with SD of 0.04. The algorithm's result was 0.67 with the same SD. The VCDRs recorded by ophthalmologists numbers one to six were 0.60, 0.61, 0.58, 0.59, 0.67, and 0.60, respectively, with SD of 0.03. The algorithm's result was 0.69, again without any change in SD. In the second image, the HCDRs recorded by ophthalmologists numbers one to six were 0.47, 0.58, 0.44, 0.56, 0.50, and 0.49, respectively, with SD of 0.06. The algorithm's result was 0.55, with SD of 0.05. On the other hand, the VCDRs recorded by ophthalmologists numbers one to six were 0.55, 0.57, 0.54, 0.59, 0.53, and 0.55, respectively, with SD of 0.02. The algorithm's result was 0.55 without any change in the SD.

**Figure 14:**
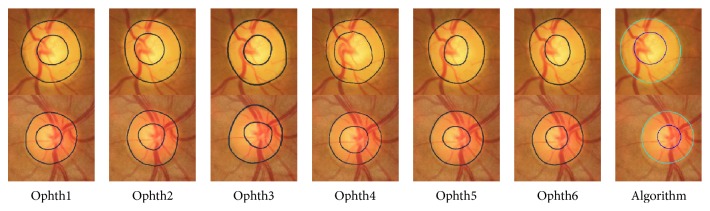
The algorithm final results for Bin Rushed images set.

Figure 14 shows the results for two images from Bin Rushed dataset. In the first image, markings by ophthalmologists numbers four and five were removed due to the cup size making the SD more than 3000 pixels. The HCDRs recorded by ophthalmologists numbers one to six were 0.53, 0.54, 0.49, 0.65, 0.43, and 0.45, respectively, with SD of 0.05. The algorithm's result was 0.53 without any change in the SD. The VCDRs recorded by ophthalmologists numbers one to six were 0.48, 0.51, 0.46, 0.61, 0.47, and 0.47, respectively, with SD of 0.06. The algorithm's result was 0.5, reducing the SD to 0.05. In the second image, the HCDRs recorded by ophthalmologists numbers one to six were 0.52, 0.53, 0.53, 0.55, 0.53, and 0.45, respectively, with SD of 0.03. The algorithm's result was 0.45, increasing the SD to 0.04. The VCDRs recorded by ophthalmologists numbers one to six were 0.49, 0.48, 0.45, 0.51, 0.43, and 0.44, respectively, with SD of 0.03. The algorithm's result was 0.045, without any change in the SD.

**Figure 15:**
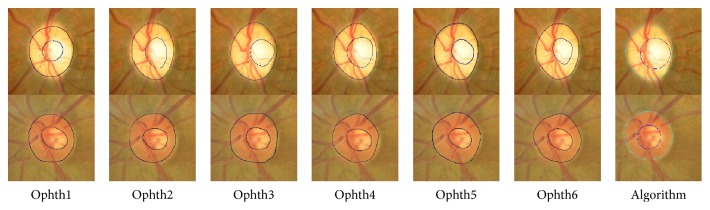
The algorithm final results for Magrabi images set.

Finally, Figure 15 shows the results of Magrabi dataset. For the first image, the top row, markings by ophthalmologists numbers one and three were removed because the SD was more than 8000 pixels for area and more than 10 pixels for centroid. The HCDRs recorded by ophthalmologists numbers one to six were 0.46, 0.52, 0.53, 0.60, 0.51, and 0.47, respectively, with SD of 0.05. The algorithm's result was 0.64, while increasing the SD to 0.06. The VCDRs recorded by ophthalmologists numbers one to six were 0.41, 0.55, 0.46, 0.54, 0.44, and 0.47, respectively, with SD of 0.05. The algorithm's result was 0.57, changing the SD to 0.055. In the second image, bottom row, the HCDRs recorded by ophthalmologists numbers one to six were 0.58, 0.54, 0.50, 0.54, 0.47, and 0.52, respectively, with SD of 0.03. The algorithm's result was 0.48, without any change in the SD. The VCDRs recorded by ophthalmologists numbers one to six were 0.47, 0.45, 0.44, 0.45, 0.39, and 0.45, respectively, with SD of 0.03. The algorithm's result was 0.049, increasing the SD to 0.035.

#### 3.3.1. Results of Bin Rushed Dataset

Table 12 illustrates the final results for Bin Rushed dataset. Seventy-three to 98 images (37.4% to 50% of the total number of images in the dataset) were eliminated due to the outliers of the aforementioned four parameters. As mentioned before, for all the parameters 10 images were not localized. The most accurate results were obtained by ophthalmologist number one (82.4% accuracy) for the total of 97 tested images. The algorithm was the second best, with 70.7% accuracy for the total of 82 tested images. Ophthalmologist number four had the least accurate results (36.6% accuracy) for the total of 112 tested images.

#### 3.3.2. Results of Magrabi Dataset

Table 13 shows the results of Magrabi dataset. From this dataset 40 to 45 images (42% to 50%) were eliminated due to outliers. The percentages of images removed due to outliers were similar for Magrabi and Bin Rushed datasets. In addition, 6 images from Magrabi dataset were not localized. The best percentage accuracy was for the algorithm (77.2%) for a total of 44 tested images.

#### 3.3.3. Results of MESSIDOR Dataset

Finally, Table 14 shows the results for MESSIDOR dataset. Sixty-nine to 86 images were removed due to the outliers. This accounts for 26% to 33% percent of images in MESSIDOR dataset which was clearly a smaller percentage in comparison with the other two datasets. In addition, 10 images were not localized. The best percentage accuracy was for ophthalmologist number three (84.7%) for a total of 164 tested images. The algorithm and ophthalmologist number one tied for the second best with 77.3% accuracy when testing a total of 168 and 172 images, respectively.

#### 3.3.4. The Final Consolidated Results

As a comprehensive analysis, Table 15 illustrates the results for all three datasets combined together for all the following four parameters: (1) optic disc, (2) optic cup, (3) HCDR, and (4) VCDR. 187 to 209 images (43 to 38% of the total images) were eliminated from the work of the six ophthalmologists due to outliers. On the other hand, 225 images (50% of the total images) were eliminated from testing the algorithm due to outliers. In addition, 26 images were not localized. The best accuracy was for markings by ophthalmologist number one (77.4% which equals 244 out of the 315 tested images). The algorithm had the second best percentage accuracy (74.2% which equals 222 out of 299 tested images). The performance of ophthalmologist number three was the third best, with 73.3% accuracy which equals 234 out of 319 tested images.

**Figure 16:**
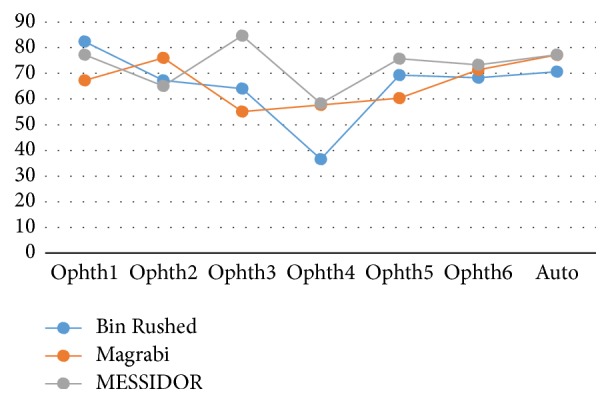
The percentage accuracy of the final results for the three datasets.

Figure 16 illustrates the variation in performance of the six ophthalmologists as well as the algorithm on the three datasets in terms of percentage accuracy. The algorithm and ophthalmologist number six showed the most consistent performance across all three datasets. The performance of ophthalmologist number two varied slightly; he performed slightly better on Magrabi dataset than on the other two datasets. Performances of ophthalmologists number one and five showed larger variations; ophthalmologist number one performed best with Bin Rushed dataset, while ophthalmologist number five performed best with MESSIDOR dataset.

Ophthalmologist number three clearly showed a better performance when working on the MESSIDOR dataset. Ophthalmologist number four performed very poorly when working on Bin Rushed dataset. Ophthalmologist numbers three, four, five, and six as well as the algorithm had the best percentage accuracy for MESSIDOR dataset. However, ophthalmologist number two had the lowest percentage accuracy when working with MESSIDOR dataset. Two ophthalmologists (#4 and 6) as well as the algorithm showed their second best performance when working on Magrabi dataset. The other three ophthalmologists (#2, 3, and 5) showed their second best performance when working on Bin Rushed dataset. Therefore, we conclude that MESSIDOR dataset was the best, followed by Magrabi dataset in the 2nd place and Bin Rushed dataset in the 3rd place.

#### 3.3.5. Agreement for the Final Consolidated Results

The best agreement in terms of the number of images was between ophthalmologists numbers one and three in 242 of the total 550 images (44%) (Table 16). The second best agreement was between ophthalmologist number one and the algorithm as well as ophthalmologist number five in 230 (41.8% of images). The agreement among these images was in all four parameters.

**Figure 17:**
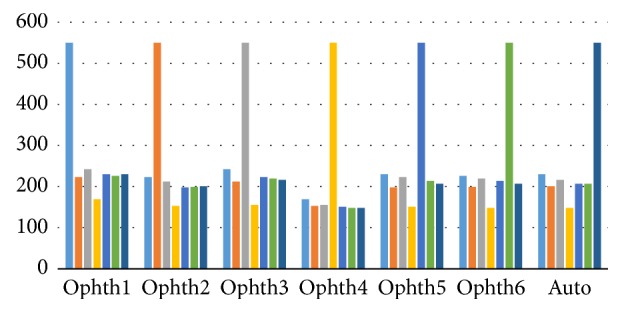
The number of images agreed for the HCDR and VCDR between the ophthalmologists and the algorithm. *x*-axis represents the number of 6 ophthalmologists and the algorithm. *y*-axis represents the number of agreed images.

Figure 17 shows the number of images agreed between the ophthalmologists and the algorithm. Clearly, the agreements among all ophthalmologists as well as the algorithm were in close to 200 images, except for ophthalmologist number four.

For all six ophthalmologists as well as the algorithm the total number of images agreed upon was in the range of 1200 to 1300 images. The only exception was ophthalmologist number four who in total had less than 1000 images agreed (Table 15).

**Figure 18:**
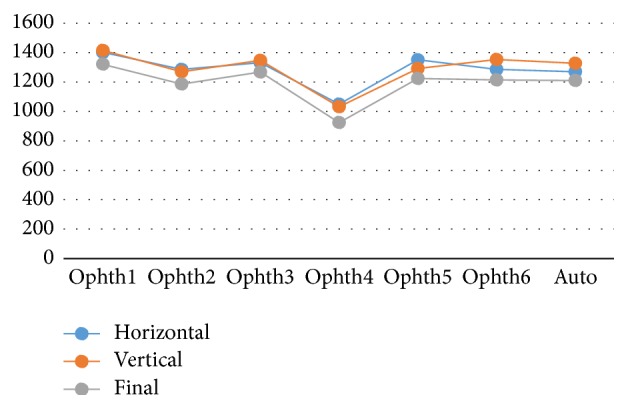
The total number of agreement for HCDR and VCDR and final consolidated results (four parameters).

Figure 18 shows that the range of the total number of images agreed for HCDR and VCDR was from 1300 to 1400 images, except for ophthalmologist number four. Clearly, the HCDR and VCDR had almost equal number of image agreements. Ophthalmologist number five had slightly more image agreements in the HCDR than VCDR, while ophthalmologist number six and the algorithm had slightly more agreed images in VCDR than HCDR. In conclusion, the VCDR was better than HCDR in terms of the total images agreed between the six ophthalmologists and the algorithm. The range of the final total image agreements including disc, cup, HCDR, and VCDR was between 1200 and 1300 images, except for ophthalmologist number four who had around 950 image agreements. The highest image agreements were for ophthalmologist number one and then ophthalmologist number three, while ophthalmologists numbers two, five, and six and the algorithm had almost the same number of image agreements.

**Figure 19:**
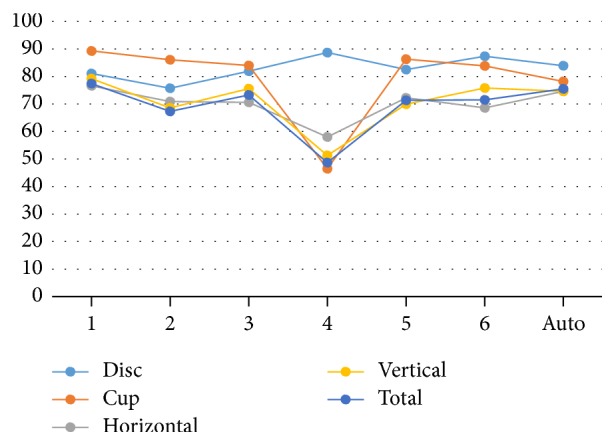
The percentage accuracy results for all the four parameters. *x*-axis represents the number of 6 ophthalmologists and the algorithm. *y*-axis represents the accuracy (percentage).

Figure 19 illustrates the percentage accuracy in marking all four parameters individually as well as the final consolidated results (all the four parameters together) for the six ophthalmologists and the algorithm. Clearly, the accuracy of disc and cup measurements influences the accuracy of HCDR and VCDR as well as the total accuracy. The cup accuracy was the best for four ophthalmologists (#1, 2, 3, and 5) due to the bigger mean SD for both area and centroid for the cup, with more accurate results for manual markings. The algorithm showed the most accurate performance when marking the disc, followed by the cup, HCDR, and VCDR. In terms of accuracy, the performance of the algorithm was similar to that of ophthalmologist number six.

Ophthalmologist number four was best in marking the disc and worst in marking the cup, with these measurements clearly affecting the HCDR and VCDR measurements.

## 4. Discussion

The goal was to have an automatic system capable of segmenting disc and cup boundaries as accurately as it was done manually. Localizing the ROI was the preprocessing step introduced in order to allow dealing with a small part of the image instead of the whole image. This was done by applying an Interval Type-II fuzzy entropy based thresholding scheme along with Differential Evolution to determine the location of the optic disc. The multilevel image segmentation was a method to segment the image into various objects in order to find the brightest object of the image, which was located at the optic cup. In terms of the main process, the optic disc segmentation was introduced first by applying the active contour implemented by level set function after inpainting the blood vessels. Inpainting was done to remove the obstacles that might be present at the level set due to the change in the intensity of the blood vessels. A double level set was applied with more processing for the low quality images (with nonmydriatic) of the Bin Rushed dataset. On the other hand, cup segmentation was conducted in two stages. In the main stage, the disc segmentation was not considered. The blood vessels were extracted in order to detect the vessel kinks to help detect the cup boundaries. The Interval Type-II fuzzy entropy based thresholding scheme and Differential Evolution were applied again on the localized image to detect the intensity of the optic cup borders. Then Hough transform was applied in order to approximate the cup. In the second stage, the disc segmentation was involved in order to improve the cup centroid accuracy by developing two more functions for *X* and *Y* coordinates.

**Figure 20:**
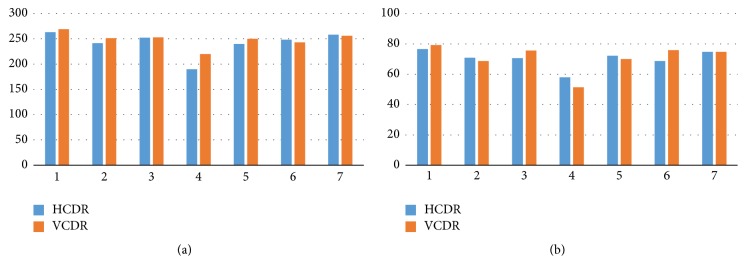
The results of HCDR and VCDR: (a) number of the accepted images (passing the image filtration process of the three parameters); (b) percentage accuracy.

After screening the images, only the successfully segmented images in terms of disc and cup were included in calculations of the HCDR. The screening process allowed including only images that met the conditions for the three parameters of disc, cup, and HCDR. The same procedures were repeated for the VCDR. Thus, in the final analysis only the images that met the conditions of the disc, cup, HCDR, and VCDR were considered. As illustrated in Figure 20(a), the algorithm had almost the same number of images segmented accurately for both HCDR and VCDR as ophthalmologist number three. Four ophthalmologists had more images accepted for the VCDR due to fewer blood vessels in these two sides (superior and inferior). However, the accepted images for the HCDR were less accurate due to the existence of blood vessels that covered the cup boundaries. One ophthalmologist had the same number of accepted images for the VCDR and HCDR as the algorithm. Therefore, the algorithm was the second best for marking the HCDR and the fourth best for marking the VCDR in terms of accuracy (Figure 20(b)).

**Figure 21:**
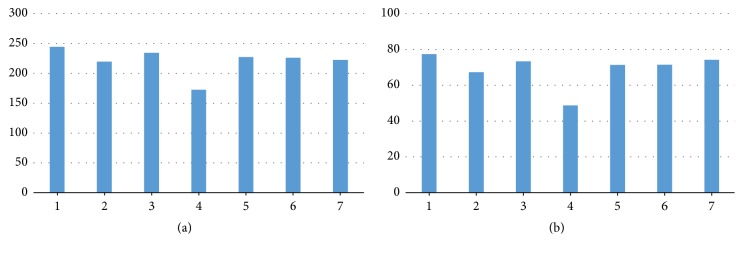
The final total results: (a) the number of accepted images (passing the image filtration process of the four parameters); (b) percentage accuracy.

Figure 21 shows the final results considering the four parameters. In terms of the number of images, the algorithm was the fifth best as illustrated in Figure 21(a). In terms of percentage accuracy, however, the algorithm was the second best due to the total number of tested images which varied among the six ophthalmologists as well as the algorithm due to removal of outliers. Around 220 to 250 images were accepted for all six ophthalmologists, except for ophthalmologist number four. The percentages of accuracy were about 70% to 80%, except for ophthalmologist number four (Figure 21(b)).

Tables 17 and 18 provide additional details about the best and worst accuracy results, and the highest and lowest number of images agreed among the six ophthalmologists. Ophthalmologist number one was the best for Bin Rushed dataset represented by the final results which considered the cup, HCDR, and VCDR (Table 16). Three ophthalmologists as well as the algorithm shared the best rank for Magrabi dataset. Ophthalmologist number three showed the best performance for analyzing MESSIDOR dataset for disc, HCDR, and VCDR. In general and considering all three datasets, ophthalmologist number one showed the best performance in analysis of the cup, HCDR, and VCDR and had the highest number of image agreements (Table 16).

Ophthalmologist number four had the worst accuracy for Bin Rushed and MESSIDOR datasets and ophthalmologist number three had the worst accuracy for Magrabi dataset (Table 17).

Finally, ophthalmologist number four had the worst percentage accuracy for the final total as well as the lowest number of images agreed with other ophthalmologists as well as the algorithm (Table 17).

We have shown that there is considerable variability among ophthalmologists in marking the fundus images Almazroa et al. [14]. Many factors, including fatigue, time of day, and concentration, may contribute to the variability in human markings. Therefore, it is important to have an “objective” means of calculating these parameters of interest to the clinician. Future work should aim at developing a smartphone application that can be used in conjunction with a fundus lens attachment to take fundus images and process them. The application can be used to examine the optic nerve head structure and help with diagnosis of glaucoma. Such system will be of great use especially in developing nations where access to tertiary or specialized centers for glaucoma care is unavailable or difficult. Such a system would be an integral part of telemedicine; the initial diagnosis is done using the smartphone application and once the cup to disc ratio is found to be indicative of glaucoma the tertiary hospital or specialist is automatically notified. The smartphone application will also allow the patient to take fundus images at home in order to closely and carefully monitor the progress/remission of the disease as well as follow the course of therapeutics. Developing a graphical user interface that allows manual modification of the algorithm (particularly for the cup) should also be considered in the future work. Such feature will allow the ophthalmologist to bring an automatically segmented cup into any position that he/she considers best suited based on his/her clinical experience. These issues will be pursued in future research.

## Figures and Tables

**Table 1:** Dataset information.

Dataset	MESSIDOR	Bin Rushed	Magrabi
Normal	Yes	Yes	Yes
Glaucomatous	Yes	Yes	Yes
Camera quality	Nonmydriatic camera (lower quality)	Nonmydriatic camera (lower quality)	Mydriatic camera (better quality)
Image size	2240 × 1488 1440 × 960	2376 × 1584	2743 × 1936
Number of training images	200	0	0
Number of testing images	260	195	95
Total images	460	195	95

**Table 2:** The HCDR results for Bin Rushed images set.

	Ophth1	Ophth2	Ophth3	Ophth4	Ophth5	Ophth6	Auto
Total number of images	195	195	195	195	195	195	195
# of images removed due to the lack of agreement among the ophthalmologists	77	67	58	58	76	76	74
Images not localized	10	10	10	10	10	10	10
Total number of images tested	108	118	127	127	109	109	111
Accuracy (number of images)	86	85	82	77	79	74	82
Accuracy (percentage)	79.6	72	64.5	60.6	72.4	67.8	73.8

**Table 3:** The HCDR results for Magrabi images set.

	Ophth1	Ophth2	Ophth3	Ophth4	Ophth5	Ophth6	Auto
Total number of images	95	95	95	95	95	95	95
# of images removed due to the lack of agreement among the ophthalmologists	40	38	35	40	35	31	43
Images not localized	6	6	6	6	6	6	6
Total number of images tested	49	51	54	49	54	58	46
Accuracy (number of images)	35	39	28	32	35	39	35
Accuracy (percentage)	71.4	76.4	51.8	65.3	64.8	67.2	76

**Table 4:** The HCDR results for MESSIDOR images set.

	Ophth1	Ophth2	Ophth3	Ophth4	Ophth5	Ophth6	Auto
Total number of images	260	260	260	260	260	260	260
# of images removed due to the lack of agreement among the ophthalmologists	56	65	73	63	67	63	64
Images not localized	10	10	10	10	10	10	10
Total number of images tested	194	185	177	187	183	187	186
Accuracy (number of images)	148	127	143	111	136	130	139
Accuracy (percentage)	76.2	68.6	80.7	59.3	74.3	69.5	74.7

**Table 5:** The HCDR results for all three images' set.

	Ophth1	Ophth2	Ophth3	Ophth4	Ophth5	Ophth6	Auto
Total number of images	550	550	550	550	550	550	550
# of images removed due to the lack of agreement among the ophthalmologists	173	170	166	161	178	170	181
Images not localized	26	26	26	26	26	26	26
Total number of images tested	351	354	358	379	346	354	343
Accuracy (number of images)	269	251	253	220	250	243	256
Accuracy (percentage)	76.6	70.9	70.6	58	72.2	68.6	74.6

**Table 6:** The number of images agreed for the HCDR between the ophthalmologists as well as the algorithm.

	Ophth1	Ophth2	Ophth3	Ophth4	Ophth5	Ophth6	Auto
Ophth1	550	238	247	192	251	234	239
Ophth2	238	550	222	173	226	214	212
Ophth3	247	222	550	177	241	224	220
Ophth4	192	173	177	550	177	168	162
Ophth5	251	226	241	177	550	233	223
Ophth6	234	214	224	168	233	550	213
Auto	239	212	220	162	223	213	550

Total	1401	1285	1331	1049	1351	1286	1269

**Table 7:** The VCDR results for Bin Rushed images set.

	Ophth1	Ophth2	Ophth3	Ophth4	Ophth5	Ophth6	Auto
Total number of images	195	195	195	195	195	195	195
# of images removed due to the lack of agreement among the ophthalmologists	78	73	72	64	69	80	76
Images not localized	10	10	10	10	10	10	10
Total number of images tested	107	112	113	121	116	105	109
Accuracy (number of images)	91	76	74	44	76	79	82
Accuracy (percentage)	85	67.8	65.4	36.3	65.5	75.2	75.2

**Table 8:** The VCDR results for Magrabi images set.

	Ophth1	Ophth2	Ophth3	Ophth4	Ophth5	Ophth6	Auto
Total number of images	95	95	95	95	95	95	95
# of images removed due to the lack of agreement among the ophthalmologists	42	40	38	44	39	41	39
Images not localized	6	6	6	6	6	6	6
Total number of images tested	47	49	51	45	50	48	50
Accuracy (number of images)	32	38	29	29	30	38	38
Accuracy (percentage)	68	77.5	56.8	64.4	60	79.1	76

**Table 9:** The VCDR results for MESSIDOR images set.

	Ophth1	Ophth2	Ophth3	Ophth4	Ophth5	Ophth6	Auto
Total number of images	260	260	260	260	260	260	260
# of images removed due to the lack of agreement among the ophthalmologists	72	60	81	62	73	76	64
Images not localized	10	10	10	10	10	10	10
Total number of images tested	178	190	169	188	177	174	186
Accuracy (number of images)	140	127	149	117	134	131	138
Accuracy (percentage)	78.6	66.8	88.1	62.2	75.7	75.2	74.1

**Table 10:** The VCDR results for all the three images sets.

	Ophth1	Ophth2	Ophth3	Ophth4	Ophth5	Ophth6	Auto
Total number of images	550	550	550	550	550	550	550
# of images removed due to the lack of agreement among the ophthalmologists	192	173	191	170	181	197	179
Images not localized	26	26	26	26	26	26	26
Total number of images tested	332	351	333	370	343	327	345
Accuracy (number of images)	263	241	252	190	240	248	258
Accuracy (percentage)	79.2	68.6	75.6	51.3	69.9	75.8	74.7

**Table 11:** The number of images agreed for the VCDR between the ophthalmologists as well as the algorithm.

	Ophth1	Ophth2	Ophth3	Ophth4	Ophth5	Ophth6	Auto
Ophth1	550	232	253	186	238	254	252
Ophth2	232	550	219	172	207	222	218
Ophth3	253	219	550	172	231	239	232
Ophth4	186	172	172	550	162	171	168
Ophth5	238	207	231	162	550	232	221
Ophth6	254	222	239	171	232	550	235
Auto	252	218	232	168	221	235	550

Total	1415	1270	1346	1031	1291	1353	1326

**Table 12:** The final results for Bin Rushed images set.

	Ophth1	Ophth2	Ophth3	Ophth4	Ophth5	Ophth6	Auto
Total number of images	195	195	195	195	195	195	195
# of images removed due to the lack of agreement among the ophthalmologists	88	87	79	73	84	87	98
Images not localized	10	10	10	10	10	10	10
Total number of images tested	97	98	106	112	101	98	82
Accuracy (number of images)	80	66	68	41	70	67	58
Accuracy (percentage)	82.4	67.3	64.1	36.6	69.3	68.3	70.7

**Table 13:** The final results for Magrabi images set.

	Ophth1	Ophth2	Ophth3	Ophth4	Ophth5	Ophth6	Auto
Total number of images	95	95	95	95	95	95	95
# of images removed due to the lack of agreement among the ophthalmologists	43	43	40	44	41	40	45
Images not localized	6	6	6	6	6	6	6
Total number of images tested	46	46	49	45	48	49	44
Accuracy (number of images)	31	35	27	26	29	35	34
Accuracy (percentage)	67.3	76	55.1	57.7	60.4	71.4	77.2

**Table 14:** The final results for MESSIDOR images set.

	Ophth1	Ophth2	Ophth3	Ophth4	Ophth5	Ophth6	Auto
Total number of images	260	260	260	260	260	260	260
# of images removed due to the lack of agreement among the ophthalmologists	78	69	86	70	81	81	82
Images not localized	10	10	10	10	10	10	10
Total number of images tested	172	181	164	180	169	169	168
Accuracy (number of images)	133	118	139	105	128	124	130
Accuracy (percentage)	77.3	65.1	84.7	58.3	75.7	73.3	77.3

**Table 15:** Results for all three datasets combined together.

	Ophth1	Ophth2	Ophth3	Ophth4	Ophth5	Ophth6	Auto
Total number of images	550	550	550	550	550	550	550
# of images removed due to the lack of agreement among the ophthalmologists	209	199	205	187	206	208	225
Images not localized	26	26	26	10	26	26	26
Total number of images tested	315	325	319	353	318	316	299
Accuracy (number of images)	244	219	234	172	227	226	222
Accuracy (percentage)	77.4	67.3	73.3	48.7	71.3	71.5	74.2

**Table 16:** The number of images agreed for the final consolidated results between the ophthalmologists as well as the algorithm.

	Ophth1	Ophth2	Ophth3	Ophth4	Ophth5	Ophth6	Auto
Ophth1	550	223	242	169	230	226	230
Ophth2	223	550	212	153	198	199	201
Ophth3	242	212	550	155	223	219	216
Ophth4	169	153	155	550	151	148	148
Ophth5	230	198	223	151	550	214	207
Ophth6	226	199	219	148	214	550	207
Auto	230	201	216	148	207	207	550

Total	1320	1186	1267	924	1223	1213	1209

**Table 17:** The best accuracy and agreement for all the four parameters and the three images sets.

	Best accuracy				Most agreement
	Bin Rushed	Magrabi	MESSIDOR	Total	
Disc	Ophth4	Ophth4	Ophth3	Ophth4	Ophth4
Cup	Ophth1	Ophth2	Ophth5	Ophth1	Ophth1
HCDR	Ophth1	Ophth2	Ophth3	Ophth1	Ophth1
VCDR	Ophth1	Ophth6	Ophth3	Ophth1	Ophth1
Final	Ophth1	Algorithm	Ophth3	Ophth1	Ophth1

**Table 18:** The worst accuracy and agreement for all the four parameters and the three images sets.

	Worst accuracy				Lowest agreement
	Bin Rushed	Magrabi	MESSIDOR	Total	
Disc	Ophth3	Ophth2	Ophth2	Ophth2	Ophth2
Cup	Ophth4	Ophth4	Ophth4	Ophth4	Ophth4
HCDR	Ophth4	Ophth3	Ophth4	Ophth4	Ophth4
VCDR	Ophth4	Ophth3	Ophth4	Ophth4	Ophth4
Final	Ophth4	Ophth3	Ophth4	Ophth4	Ophth4

## Abbreviations

HCDR: Horizontal cup to disc ratio
VCDR: Vertical cup to disc ratio
RIGA: Retinal fundus images for glaucoma analysis
IOP: Intraocular pressure
Ophth: Ophthalmologist.
